# Mapping QTLs conferring salt tolerance and micronutrient concentrations at seedling stage in wheat

**DOI:** 10.1038/s41598-017-15726-6

**Published:** 2017-11-15

**Authors:** Babar Hussain, Stuart James Lucas, Levent Ozturk, Hikmet Budak

**Affiliations:** 1 Faculty of Engineering and Natural Sciences, Sabanci University, Istanbul, Turkey; 2 SU Nanotechnology Research and Application Centre, Sabanci University, Istanbul, Turkey; 3 Cereal genomics Lab, Department of Plant Sciences and Plant Pathology, Montana State University, Bozeman, MT USA

**Keywords:** Genetic linkage study, Plant genetics

## Abstract

Soil salinization and degradation is one of the consequences of climate change. Identification of major salt tolerance genes and marker assisted selection (MAS) can accelerate wheat breeding for this trait. We genotyped 154 wheat F_2_ lines derived from a cross between salt tolerant and susceptible cultivars using the Axiom Wheat Breeder’s Genotyping Array. A high-density linkage map of 988 single nucleotide polymorphisms (SNPs) was constructed and utilized for quantitative trait loci (QTL) mapping for salt tolerance traits and mineral concentrations under salinity. Of 49 mapped QTLs, six were for Na^+^ exclusion (NAX) and two QTLs (qSNAX.2 A.1, qSNAX.2 A.2) on chromosome 2 A coincided with a reported major NAX QTL (*Nax1* or *HKT1;4*). Two other major NAX QTLs were mapped on 7 A, which contributed 11.23 and 18.79% of the salt tolerance respectively. In addition to Ca^+2^ and Mg^+2^ QTLs, twenty-seven QTLs for tissue Phosphorus, Zinc, Iron, Manganese, Copper, Sulphur and Boron concentrations under salinity were also mapped. The 1293 segregating SNPs were annotated/located within genes for various ion channels, signalling pathways, transcription factors (TFs), metabolic pathways and 258 of them showed differential expression *in silico* under salinity. These findings will create new opportunities for salt tolerance breeding programs.

## Introduction

The effects of climate change are predicted to reduce the cultivated land area of the world by 2–9%^1^. This land loss or soil degradation is feared to be increased by soil salinization, which is caused both by natural processes and human activities such as saline irrigation and land clearing^1,2^. More than 800 million hectares of land including 20% of irrigated area worldwide is affected^3,4^. Soil salinity significantly reduces wheat growth and development at the seedling stage, resulting in lower grain yield as higher Na^+^ influx causes toxicity and disrupts leaf function^5^. Conversely, 100–110% extra food will be required by 2050 to feed the growing world population^6^. Therefore, utilization of saline soils through development of salt tolerant and/or climate resilient wheat is important for meeting increasing food demand.

However, despite a major focus on drought, comparatively little work has been performed on breeding wheat for salt tolerance^7^. Development of salt tolerant cultivars is hindered greatly by the complexity and severity of salt stress, which occurs in two phases, i.e. osmotic stress and ionic stress^2^. Osmotic stress, resulting from higher salt concentrations outside the root, inhibits water uptake, cell expansion and development^7^. Subsequently, high Na^+^ ion uptake into leaves promotes leaf chlorosis, necrosis and mortality due to reduced photosynthesis^2,7^. Wheat yield data collected from field experiments cannot easily be used as a salt tolerance index due to the range of interactions between variable Na^+^ in soil profile, differential salt responses depending on genotype, growth stage, and other factors such as high pH and drought^7^. However, hydroponics/pot screening performed in greenhouse conditions and physiological studies have indicated that wheat has significant genetic variation for salt tolerance^4,8,9^ which can be exploited for wheat breeding and genetics. There are few works has been directed to exploring the physiological and genetic complexity of multi-genic and multi-faceted salinity related traits.

Recent development in genomic knowledge and technology has provided new horizons and foundations for genetic improvement of complex traits such as drought and salt tolerance. The combination of genomic tools with MAS can be used to identify and select the preferred genes in breeding populations at a much faster rate than by classical breeding^10–15^. Until recently, progress in MAS for wheat was slowed by the limited availability of genomic data, but advances in genotyping techniques and DNA sequencing have now produced genome datasets that have been used to design sequence-based simple sequence repeats (SSRs) and SNP markers^16–18^. Among the various marker types, SNPs are increasingly used for germplasm characterization and gene mapping as they provide cost-effective, rapid and high-throughput genotyping^18^. As SNPs are co-dominant, sequence tagged and highly abundant, they are suitable for the dissection of complex traits using highly multiplexed marker microarrays such as the Affymetrix GeneChip^19^. For example, the Axiom Wheat Breeders’ Genotyping array is a recently developed, highly efficient system for screening large wheat populations. It contains 35,143 pre-validated SNPs which cover all 21 wheat chromosomes and is capable of genotyping 384 wheat samples simultaneously, making it a cost-effective high-throughput genotyping method. Recently, it has also been used for construction of a high-density linkage map and subsequent identification of genomic regions for drought-related traits in durum wheat^20^.

Genotipic data from multiplexed marker assays is used to construct high-density linkage maps, which is a prerequisite to mapping the QTLs for agronomically important traits^21–24^. High-density linkage maps also provide a genomic resource for positional cloning of important genes. Due to their construction from sequenced-tagged markers, they can also be used for comparative genomics to dissect chromosomal organization and evolution^21^. Markers in the linkage map provide tags for regions containing QTLs of targeted traits, and several QTLs for salt tolerance have previously been mapped in wheat^23,25,26^. Genc *et al*.^25^ mapped 40 QTLs for seven seedling traits including chlorophyll content, Na^+^ and K^+^ concentrations in shoot, and seedling biomass under salinity. A NAX QTL interval i.e. wPt-3114-wmc170, mapped on Chromosome 2A, was associated with 10% increase in seedling biomass. Two of the five QTLs for NAX were co-located with seedling biomass QTLs, however, all five QTLs contributed only 18% of the seedling biomass phenotypic variation. Therefore, a need remains for QTL mapping for salt tolerance in more populations to identify other major/novel QTLs.

It was suggested that in addition to NAX and K^+^, several other factors might be involved in wheat salt tolerance. For example, it has been reported that Mg^2+^ and Ca^2+^ accumulation also influence salt tolerance and several QTLs for Mg^2+^ and Ca^2+^ concentrations under salinity were identified in wheat^27^. Apart from K^+^, Mg^2+^ and Ca^2+^, genetic bases of other minerals such as P, Zn, Fe, Mn, Cu, S and Boron has been investigated under different water regimes, but not under salinity^24^. Therefore, we aimed to identify the genetic basis of accumulation of these minerals under salt stress. Additionally, we also report the functional annotation of linkage-mapped SNPs for an F2 population de for salt tolerance.

The objectives of the present study were: (a) construction of a high-density SNP linkage map for an F_2_ population with phenotypic variation for salt tolerance, (b) identification of QTLs and markers linked with various micronutrient concentrations and salt tolerance related seedling traits, and (c) functional annotation of segregating markers in the F_2_ population, and (d) validation of annotated SNPs/genes by differential expression analysis.

## Materials and Methods

### Plant material

The F_2_ population was developed from hybrids of bread wheat cultivars, with contrasting salt tolerance (WTSD91 and WN-64). WTSD91 and WN-64 were found to be moderately salt tolerant and highly susceptible respectively, under 300 mM Sodium Chloride (NaCl) treatment in hydroponics screening^4^. Crossed seeds were grown during 2012–13 to generate F_1_ hybrids. To ensure purity, heads were covered with paper bags during anthesis, and seeds for F_2_ lines were obtained.

### Growth conditions and phenotyping

The experiment was conducted in Venlo-type greenhouse located at 40°53′25″N, 29°22′47″E in Istanbul, equipped with computerized climate control for supplemental lighting, evaporative cooling and heating. The day and night temperatures were kept at 25 ± 4 °C and 20 ± 4 °C during the experiment period. 250 F_2_ lines were germinated in perlite for 5 days and 180 seedlings were then transplanted into 2.7-L pots containing aerated nutrient solution^28^ after removing their residual endosperm. On the following day, 75 mM NaCl was added to the nutrient solution. The solution was replaced every four days and the salinity level increased successively to 150, 225 and 300 mM NaCl on the 4^th^, 8^th^ and 12^th^ day after transplantation. Plants remained under salinity for a total of 32 days including 20 days at 300 mM NaCl. Plants were then divided into four groups based on their phenotype: (i) tolerant (T) group had 5 fully extended green leaves without any signs of salt injury; (ii) moderately tolerant (MT) group had 4–5 fully extended green leaves with minor salt injury signs on leaf tips; (iii) susceptible (S) group had 2–3 leaves showing severe salt injury signs and 1–2 dead leaves; and (iv) highly susceptible (HS) group with 2–3 leaves having severe injury signs and death of 60–80% of leaves. Four representative plants from each group were selected for mineral analysis.

After three washings in dH_2_O, shoots and roots were dried at 65 °C for 3 days, dry root weight (DRW) and dry shoot weights (DSW) were recorded and tissues analyzed for mineral concentrations as previously described^28^. Dry roots and shoots were ground to powder in an agate vibrating cup mill (Pulverisette 9; Fritsch GmbH; Germany) and ~0.15–0.2 g powder from each sample was digested in 5 ml of 65% HNO_3_ and 2 ml of 30% H_2_O_2_ using closed-vessel microwave system (Mars Express; CEM Corp; NC, USA). Digested solutions were diluted with milli-Q water to 20 ml final volume for measurement of Ca, Cu, Fe, K, Mg, Mn, P, S, Zn and Boron concentrations by inductively coupled plasma optical emission spectrometry (ICP-OES; Vista-Pro Axial; Varian Pty Ltd; Mulgrave, Australia)^29^. The mineral concentration data was checked against standard values for standard reference material (SRM 8436 Durum Wheat Flour, NIST, Gaithersburg, MD). For measuring Na^+^ concentration, solutions were further diluted 1:50. The data for 22 traits was obtained by multiplying ICP-OES values by the dilution factor and dividing the result by the DRW or DSW used for digestion. As reduction in the concentration of Na^+^ means higher NAX so Na^+^ values were multiplied by −1 to get root Na exclusion (RNAX) and shoot Na exclusion (SNAX) values. Correlation coefficients among the phenotypic data were calculated using Statistix 8.1 program.

### DNA extraction and genotyping

DNA extraction of 164 F_2_ lines and the two parents was carried out using the Wizard Genomic DNA Purification Kit (Promega, Madison, WI, USA). DNA concentrations were measured with the Quant-iT PicoGreen dsDNA Assay Kit (ThermoFisher Scientific, Waltham, MA, USA) and 1.5 µg of gDNA from each line was dissolved in 10 mM Tris-HCl pH 8.0 to a final volume of 30 µl. Genotyping was performed using the Axiom Wheat Breeder’s Genotyping Array (Affymetrix, Santa Clara, CA, USA) with 35,143 SNPs for each sample (hereafter referred to as the wheat 35 K array). Genotyping was done at the Bristol Genomics Facility (Bristol University, UK) utilizing the Affymetrix GeneTitan MT system following the Affymetrix procedure (Axiom 2.0 Assay Manual). We used Axiom Analysis Suite 1.1.0.616 software for SNP calling by following the Axiom Best Practices Genotyping Workflow with default wheat SNP call rate cut-off = 97%, QC call rate cut-off = 92% and DQC cut-off = 0.82. However, 10 lines failed the QC and DQC cut-offs; therefore, SNP call codes for the remaining 154 lines were used for downstream analysis. This software uses cluster separation, call rate and deviation from expected cluster positions and classifies the SNPs into six performance categories^19^.

### Construction of SNP linkage map

The markers with significant segregation distortion (P < 0.05) were removed through chi-square test combined with sequential Bonferroni correction^30^. A genetic linkage map was constructed using MapDisto 2.0 b93^22^. In MapDisto, the markers were grouped with a recombination fraction of 0.3 and logarithm of the odds ratio (LOD) score of six using the Kosambi mapping function. The Seriation algorithm was used for ordering the linkage groups. Linkage groups were assigned to chromosomes by comparing shared markers with a recent wheat consensus linkage map^19^. Chromosomes were found to be divided into multiple linkage groups, so these linkage groups were combined and re-ordered. Rippling of marker order with a window size of five markers and checking for inversions were performed to improve the marker order and produce the shortest map of each chromosome.

### QTL mapping

Additive QTLs for all 22 traits were mapped by the composite interval mapping (CIM) method using single-treatment phenotypic data and QTL IciMapping V4.1.0 with 1-cM walking speed and LOD thresholds of 2.5^31^. The linkage maps and QTLs were drawn using MapChart 2.30 software^32^. The contribution of single QTL in phenotypic variation of mineral concentrations and salt tolerance was calculated by following Zhang *et al*.^33^. The DRW and DSW are considered to be the direct measure of salt tolerance^25,34^ so the contribution of QTLs to salt tolerance was calculated by using DRW and DSW data.

### Functional annotation of segregating SNPs

The flanking sequences of 3381 ‘Poly High Resolution’ (PHR) or polymorphic SNPs were mapped to gene coding sequences (CDS) from the International Wheat Genome Sequencing Consortium (IWGSC) database^35^ using BLAST+ 2.2.30. From 1448 initial hits, basic local alignment search tool (BLAST) alignments with less than 60 bp length and/or 95% identity were removed. In this way, 1323 SNPs were found to be located in 1257 IWGSC CDS which were functionally annotated by using Blast2GO V4.0^36^. Functional annotation was done by using NCBI blast, mapping and annotation commands of Blast2Go using default parameters, and annotations with E-value ≥1 × 10^−30^ were discarded.

### *In-silico* expression analysis of annotated genes

For the purpose, above mentioned 1257 IWGS CDS were aligned with transcriptome reads expressed under normal and saline conditions^3^ by using BLASTN 2.6.1+. Significant alignments with >200 alignment scores were counted and salt/normal alignment counts ratio was divided by 3.25 (347,200/106,600 spots for salt/control) to get the expression value. Genes with at least a 2-fold change in expression up or down were considered to be differentially regulated.

## Results

### SNP calling yielded six categories of SNPs

**Figure 1:**
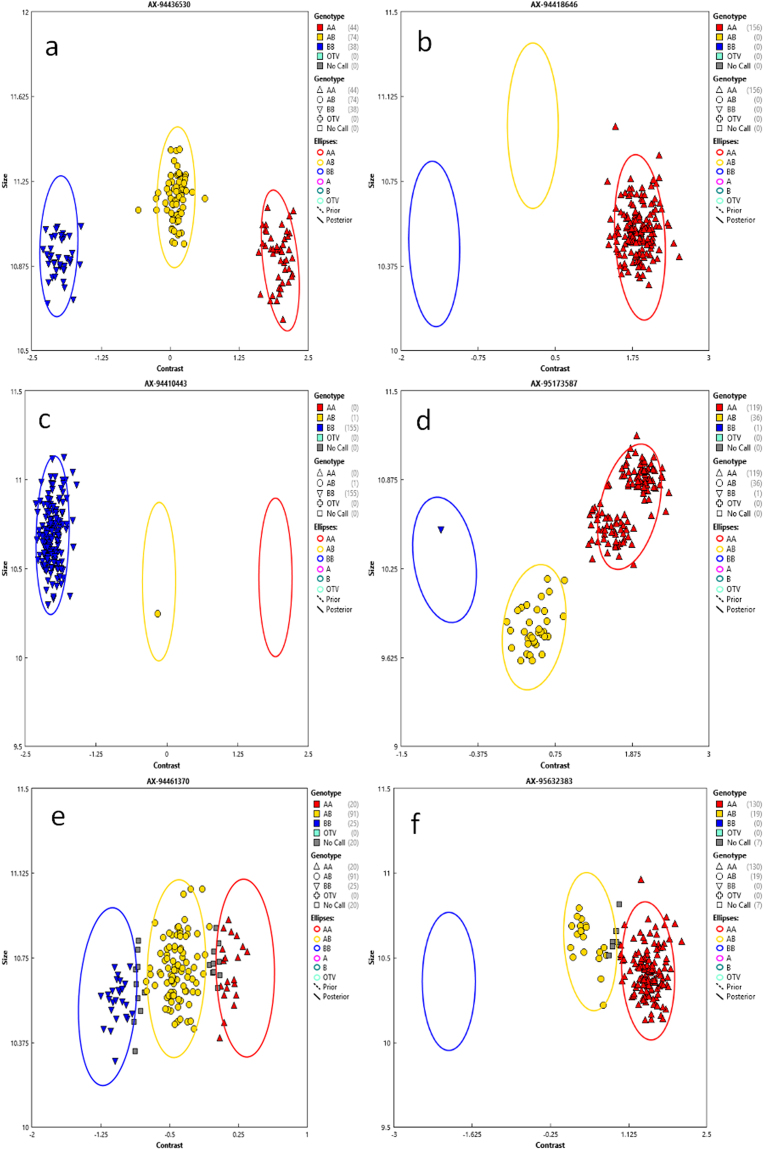
Representative of six SNPs calling categories: (**a**) Poly High Resolution; (**b**) Mono High Resolution; (**c**) No Minor Homozygote; (**d**) Off-Target Variants (**e**) Call Rate Below Threshold; and (**f**) Other.

From 164 F_2_ lines analysed on the wheat 35 K array, 154 gave good quality data and were used for SNP clustering. The SNPs were categorized into six groups: (i) PHR SNPs, which were polymorphic and co-dominant with a minimum of two samples containing the minor allele; (ii) Mono High Resolution (MHR) or monomorphic SNPs had only one cluster/allele; (iii) No Minor Homozygote (NMH), these polymorphic and dominant SNPs had only two clusters, one being the heterozygote; (iv) Off-Target Variants (OTV) had four clusters including one for a null allele; (v) Call Rate Below Threshold (CRBT) had all cluster properties above the threshold except for the call rate cut-off; and (vi) Other type SNPs, which had one or more cluster properties below quality thresholds (Fig. 1). Out of 35,143 array SNPs, 46.1% or 16,210 were MHR/monomorphic and 51 (0.15%) SNPs were OTVs. Meanwhile 3,381 (9.6%) SNPs were PHR/polymorphic (Table 1) whose call codes were used for linkage map construction.

**Table 1:** SNP calling distribution for 154 bread wheat F2 lines identified using thewheat 35 K Array.

SNPs calling categories	No. of Markers	Percent SNPs calling (%)
Mono High Resolution	16210	46.1
Poly High Resolution	3381	9.6
Other	8141	23.2
No Minor Homozygote	3017	8.6
Call Rate Below Threshold	4343	12.35
OTV	51	0.15
**Total**	**35143**	**100**

### Whole genome wheat genetic linkage map

**Figure 2:**
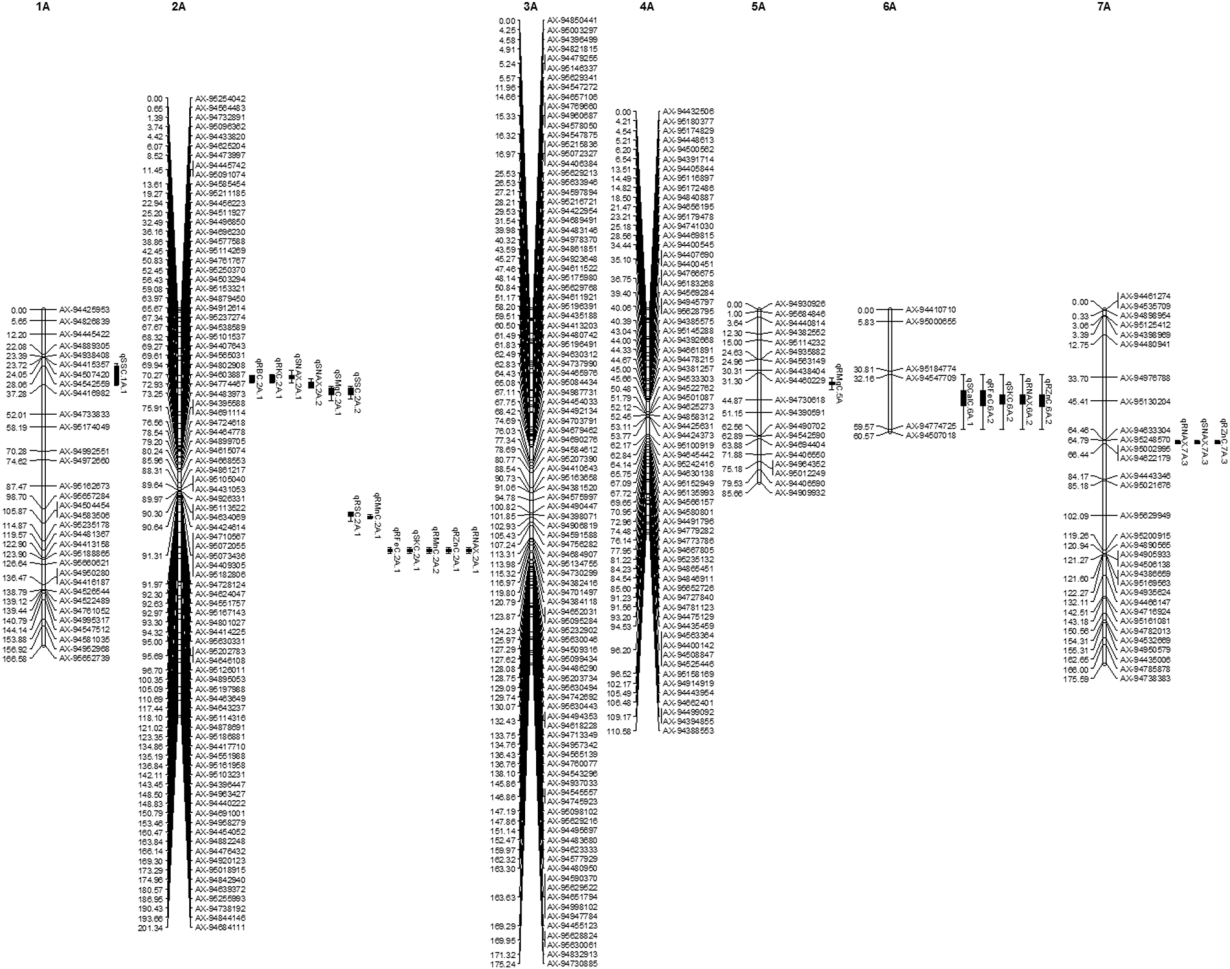
Additive QTLs mapped on A genome for salt tolerance traits and mineral concentrations under salt stress in wheat F_2_ population.

**Figure 3:**
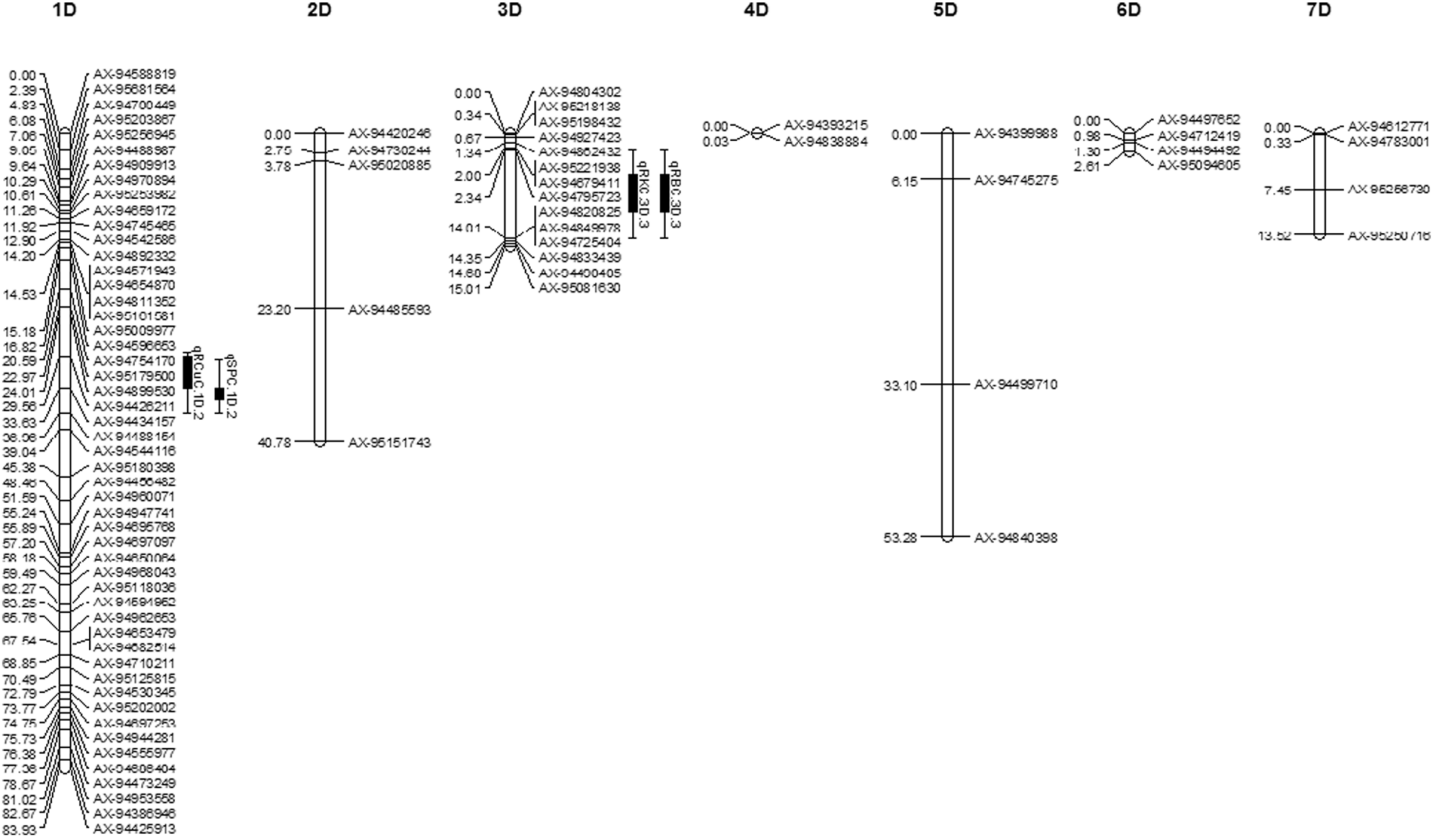
Additive QTLs mapped on B genome for salt tolerance traits and mineral concentrations under salt stress in wheat F_2_ population.

**Figure 4:**
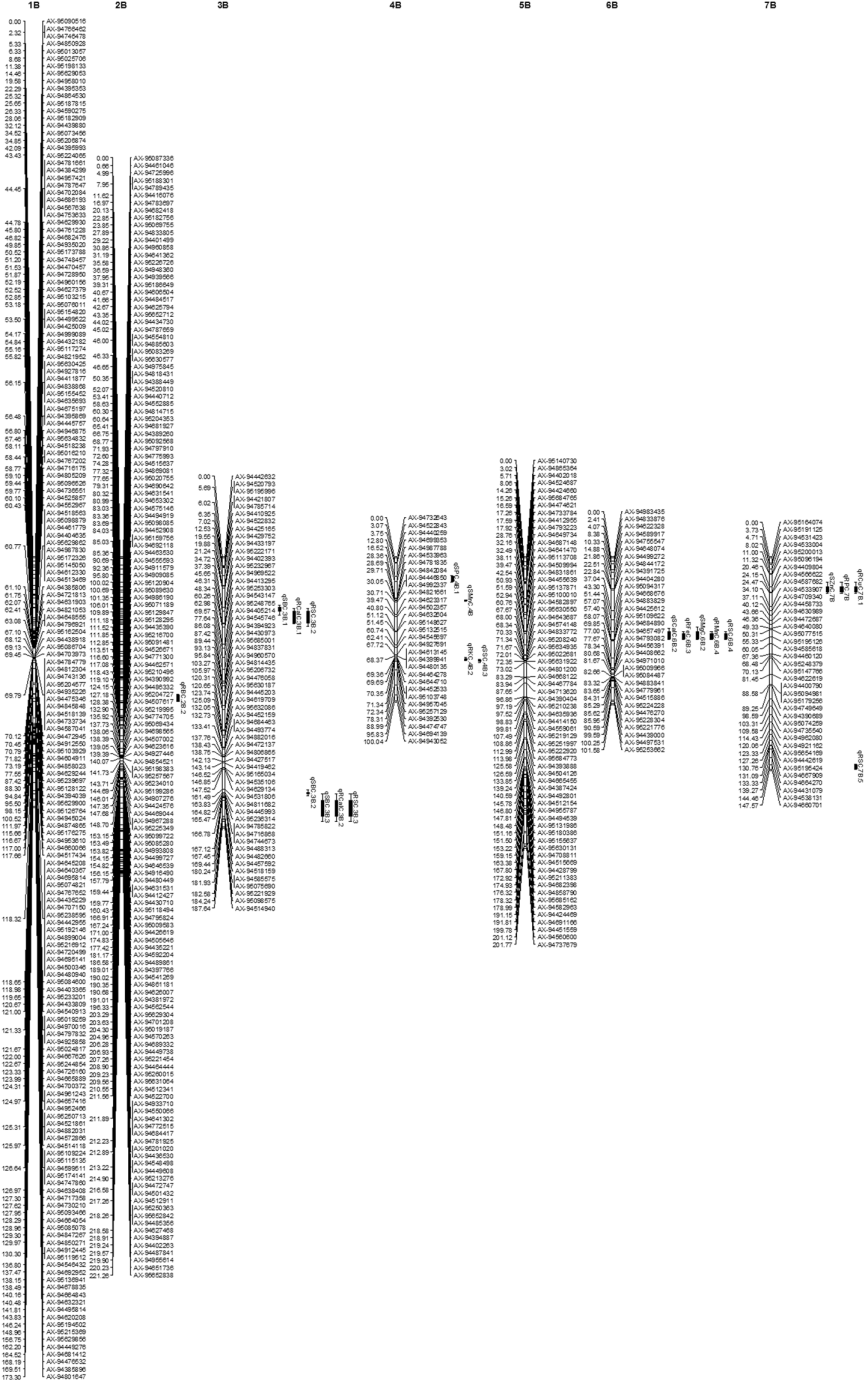
Additive QTLs mapped on D genome for salt tolerance traits and mineral concentrations under salt stress in wheat F_2_ population.

Out of 3,381 PHR markers, 1072 passed the chi-square test for segregation distortion and were used to construct the linkage map. We assigned 988 markers to 21 chromosomes and 84 markers were unlinked. The B genome had the highest number of markers assigned (562) followed by the A (342) and D (84) genomes. 183 markers were assigned to chromosome 1B while the fewest in the B genome (31) were assigned to 4B. For the A genome, maximum (100) and minimum (6) markers were assigned to chromosomes 3A and 6A respectively. In the D genome, chromosome 1D had 51 linked markers while 4D had only 2. The total length of the whole genome map was 2317.88 cM consisting of 975.56, 1133.16 and 209.16 cM for A, B and D genomes respectively. The maximum length was 221.26 cM for chromosome 2B and minimum was 0.03 cM for chromosome 4D. Average length per marker was 3.71, 4.43, 2.68 and 4.03 cM for whole genome, A, B and D genome (Table 2; Figs 2, 3 and 4).

**Table 2:** Distribution of 988 assigned markers to 21 wheat linkage groups and chromosomes lengths for an F_2_ population of 154 lines.

Chromosome	No. of Markers	Chromosome length (cM)	Length/ marker (cM)	Consensus map lengths (cM)^19^
1A	32	166.58	5.21	182.07
2A	88	201.34	2.29	203.99
3A	100	175.24	1.75	136.11
4A	66	110.58	1.48	75.68
5A	19	85.66	4.51	221.39
6A	6	60.57	10.1	189.4
7A	31	175.59	5.66	231.64
**A Genome**	**342**	**975.56**	**4.43**	**1240.28**
1B	183	173.30	0.95	182.35
2B	151	221.26	1.47	216.96
3B	59	187.64	3.18	234.56
4B	31	100.04	3.23	76.67
5B	66	201.77	3.06	208.75
6B	33	101.58	3.08	165.99
7B	39	147.57	3.78	279.28
**B Genome**	**562**	**1133.16**	**2.68**	**1364.56**
1D	51	83.93	1.65	151.29
2D	5	40.78	8.16	177.47
3D	14	15.01	1.07	234.87
4D	2	0.03	0.014	162.07
5D	4	53.28	13.32	167.57
6D	4	2.61	0.65	167.78
7D	4	13.52	3.38	73.34
**D Genome**	**84**	**209.16**	**4.03**	**1134.39**
**Total**	**988**	**2317.88**	**3.71**	**3739.23**

### Comparison of linkage map with consensus map

By comparing our map with a recently published consensus map using these markers^19^, it was found that a total of 511 of 988 (51.7%) markers were assigned to the same chromosome as consensus map and 398 of 988 or 40.28% of the SNPs were mapped for the first time. The number of markers newly assigned to the A, B and D genomes was 132, 247 and 19 respectively. The maximum of these markers in the A genome i.e. 45 were located on chromosome 2A while 1B harboured 153 of these markers (Table S1). Remaining 79 markers were assigned to different chromosomes from the consensus map. The biggest such group consisted of 32 markers assigned to chromosome 2B in our map. While 17 such markers were assigned to chromosome 2A and 29 of total 79 SNPs were assigned to their respective homoeologous chromosomes (Table S2).

### QTL mapping for salt tolerance and mineral nutrient concentrations

The phenotypic data for 22 traits and correlation coefficients among different traits are presented in Table S3 and S4 respectively. We identified 49 additive QTLs for 20 single-treatment phenotypic traits on 12 wheat chromosomes. These QTLs were mapped on two chromosomes of the D genome and 5 chromosomes each from the A and B genomes (Table 3; Figs 2, 3 and 4). Among the six QTLs detected for RNAX and SNAX, the QTL on 7A (qRNAX.7 A.3 and qSNAX.7A.3) contributed 13.69 and 15.35% of phenotypic variation of NAX from root and shoot. They contributed 11.23 and 19.79% of DRW and DSW (salt tolerance) respectively. Other RNAX and SNAX QTLs were located on 2A and 6A. Shoot K^+^ concentration (SKC) and root K^+^ concentration (RKC) QTLs were mapped on 2A, 6A, 4B and 3D, and the most important SKC QTL on 6A contributed 7.46 and 9.76% in K concentration and salt tolerance. Among the four root Zinc Conc. (RZnC) and shoot Zinc Conc. (RZnC) QTLs, RZnC QTL on 7A contributed 12.08 and 11.23% of Zn phenotype and salt tolerance respectively. For root Ca^2+^ concentration (RCalC) and shoot Ca^2+^ concentration (SCalC), QTLs qRCalC.6B.3 and qSCalC.6B.2 presented 10.91 and 6.52% of Ca conc. in wheat root and shoot. These also contributed 5.92 and 11.87% of salt tolerance. Among Mg^2+^ concentration (RMgC and SMgC) QTLs, 2A QTL contributed maximum 6.37% of phenotypic variation of SMgC and qSMgC.6B.2 contributed maximum 8.36% to salt tolerance (DSW).

**Table 3:** Additive QTLs mapped on 12 chromosomes for various mineral concentrations under 300 mM NaCl salt condition.

Trait	QTL	Marker Interval	Position (cM)	LOD	PQCMC	PQCST
RBC	qRBC.2A.1	AX-94496850–AX-94696230	32.49–36.16	3.59	1.29	0.14
	qRBC.2B.2	AX-94909085–AX-95120904	95.80–100.2	2.50	0.16	0.10
	qRBC.3D.3	AX-94795723–AX-94820825	2.34–14.01	5.68	2.29	0.21
RCalC	qRCalC.3B.1	AX-95232967–AX-94969522	37.39–45.65	2.86	5.27	0.68
	qRCalC.3B.2	AX-94457592–AX-94518159	169.44–180.24	2.84	5.38	0.98
	qRCalC.6B.3	AX-94668676–AX-94883829	51.44–57.07	7.09	10.91	5.92
RCuC	qRCuC.7B.1	AX-94409804–AX-94566622	20.46–24.15	2.75	2.83	0.08
	qRCuC.1D.2	AX-94426211–AX-94434157	29.56–33.63	2.53	6.06	0.39
RFeC	qRFeC.2A.1	AX-95114316–AX-94878691	118.10–121.02	2.51	3.98	3.81
	qRFeC.6A.2	AX-94547709–AX-94774725	32.16–59.57	26.7	12.96	3.15
	qRFeC.6B.3	AX-94668676–AX-94883829	51.44–57.07	18.10	8.73	5.92
RKC	qRKC.2A.1	AX-94496850–AX-94696230	32.49–36.16	3.70	4.79	0.14
	qRKC.4B.2	AX-95103748–AX-94957045	70.35–71.34	21.4	11.31	1.40
	qRKC.3D.3	AX-94795723–AX-94820825	2.34–14.01	4.01	7.96	0.21
RMgC	qRMgC.5 A	AX-94460229–AX-94730618	31.30–44.87	6.20	4.96	5.58
RMnC	qRMnC.2A.1	AX-94895053–AX-95197988	100.35–105.09	5.96	8.13	0.37
	qRMnC.2 A.2	AX-95114316–AX-94878691	118.10–121.02	3.01	5.17	3.81
	qRMnC.6B.3	AX-94668676–AX-94883829	51.44–57.07	19.20	14.16	5.92
RNAX	qRNAX.2A.1	AX-95114316–AX-94878691	118.10–121.02	2.53	4.85	3.81
	qRNAX.6A.2	AX-94547709–AX-94774725	32.16–59.57	9.35	6.46	3.15
	qRNAX.7 A.3	AX-95248570–AX-95002995	64.79–66.44	2.51	13.69	11.23
RPC	qRPC.7B	AX-94409804–AX-94566622	20.46–24.15	2.59	2.73	0.08
RSC	qRSC.2A.1	AX-94895053–AX-95197988	100.35–105.09	3.50	5.65	0.37
	qRSC.3B.2	AX-95232967–AX-94969522	37.39–45.65	2.96	4.61	0.68
	qRSC.3B.3	AX-94457592–AX-94518159	169.44–180.24	2.92	6.42	0.98
	qRSC.6B.4	AX-94668676–AX-94883829	51.44–57.07	16.18	10.04	5.92
	qRSC.7B.5	AX-94538131–AX-94660701	144.46–147.7	2.52	2.89	1.97
RZnC	qRZnC.2A.1	AX-95114316–AX-94878691	118.10–121.02	2.83	5.25	3.81
	qRZnC.6A.2	AX-94547709–AX-94774725	32.16–59.57	11.22	7.45	3.15
	qRZnC.7 A.3	AX-95248570–AX-95002995	64.79–66.44	2.52	12.08	11.23
SBC	qSBC.3B.1	AX-94402393–AX-95232967	34.72–37.39	2.70	5.86	0.59
	qSBC.3B.2	AX-94811682–AX-94445993	163.83–164.82	2.63	5.53	0.46
	qSBC.3B.3	AX-94457592–AX-94518159	169.44–180.24	2.82	4.91	1.01
SCalC	qSCalC.6A.1	AX-94547709–AX-94774725	32.16–59.57	29.1	8.98	3.08
	qSCalC.6B.2	AX-94668676–AX-94883829	51.44–57.07	11.41	6.52	11.87
SKC	qSKC.2A.1	AX-95114316–AX-94878691	118.10–121.02	2.51	4.34	5.18
	qSKC.6A.2	AX-94547709–AX-94774725	32.16–59.57	9.15	7.46	9.76
SMgC	qSMgC.2A.1	AX-94577588–AX-95114269	38.86–42.45	2.79	6.37	1.23
	qSMgC.6B.2	AX-94668676–AX-94883829	51.44–57.07	2.58	5.90	8.36
SMnC	qSMnC.4B	AX-94842084–AX-94446850	29.71–30.05	4.75	3.12	1.03
SNAX	qSNAX.2A.1	AX-94496850–AX-94696230	32.49–36.16	2.89	5.14	0.95
	qSNAX.2A.2	AX-94696230–AX-94577588	36.16–38.86	3.10	7.10	1.45
	qSNAX.7 A.3	AX-95248570–AX-95002995	64.79–66.44	2.92	15.35	18.79
SPC	qSPC.4B.1	AX-94699353–AX-94987788	12.80–16.52	3.38	2.10	1.62
	qSPC.1D.2	AX-94434157–AX-94488154	33.63–3696	2.58	4.73	0.20
SSC	qSSC.1 A.1	AX-94542559–AX-94416982	28.06–37.28	2.55	1.38	0.96
	qSSC.2A.2	AX-94577588–AX-95114269	38.86–42.45	4.13	2.13	0.91
	qSSC.4B.3	AX-94957045–AX-95257129	71.34–72.34	4.55	2.46	0.01
SZnC	qSZnC.7B	AX-94409804–AX-94566622	20.46–24.15	2.78	3.28	0.13

**QTL:** quantitative trait loci, **LOD:** logarithm of the odds ratio; **PQCMC:** percent QTL contribution for mineral concentration; **PQCST:** percent QTL contribution for salt tolerance.

Although no QTL was detected for shoot Cu and Fe conc. (SCuC and SFeC), a root Cu conc. (RCuC) QTL on 1D and root Fe Conc. (RFeC) QTL on 6A contributed 6.06% and 12.96% of Cu and Fe phenotypic variation. Another RFeC QTL on 6B contributed 5.92% to salt tolerance. In the same region, a QTL for Mn contributed 14.16% of Mn phenotypic variation and 5.92% of salt tolerance. Although the QTLs for Boron, P and S contributed as much as 10.04% to mineral concentration variation, they made little contribution to salt tolerance (Table 3). We found five QTL clusters, on chromosomes 2A, 6A, 7A, 3B and 6B (Figs 2 and 3).

### Candidate gene identification for segregating SNPs

Out of 3381 polymorphic SNPs, 1306 were functionally annotated based on 20 BLAST alignments for each SNP; the best/top BLAST alignments for these SNPs were associated with sequences from 22 species. With 480 homologous sequences, *Aegilops tauschii* had the maximum number of BLAST hits, whereas only 1 BLAST hit was retrieved for nine species. While 290, 280, 136 and 77 coding sequences containing SNPs were homologous to sequences from *Triticum urartu*, *Hordeum vulgare*, *T*. *aestivum* and *Brachypodium distachyon*, respectively (Table 4).

**Table 4:** Top BLAST hit distribution of 1306 wheat sequences/SNPs in 22 different species by Blast2GO alignment.

Species	BLAST top hits	Species	BLAST top hits
*Aegilops tauschii*	480	*O*. *brachyantha*	3
*Triticum urartu*	290	*Zea mays*	2
*Hordeum vulgare*	280	*B*. *sylvaticum*	1
*T*. *aestivum*	136	*Gossypium hirsutum*	1
*Brachypodium distachyon*	77	*Phyllostachys edulis*	1
*T*. *durum*	9	*P*. *praecox*	1
*Sorghum bicolor*	6	*Secale cereale*	1
*Oryza sativa Japonica*	5	*Zootermopsis nevadensis*	1
*Setaria italic*	3	*Agropyron mongolicum*	1
*T*. *monococcum*	3	*Avena longiglumis*	1
*Dichanthelium oligosanthes*	3	*A*. *sativa*	1
**Total Species**	**22**	**Total Annotations**	**1306**

Annotated sequences carrying polymorphic SNPs varied widely in function. For instance, 44 SNPs were annotated to ion transporters/channels linked to wheat salt tolerance, including seven potassium, two Chloride, four calcium, five zinc, four magnesium, two sulfate, three nitrate transporters/channels along with one proton pump, four proton transporters, cationic, anionic and metal channels (Table 5, Table S5). Another 50 SNPs were annotated for protein, glucose, malate, drug, fatty acid and proline transport along with endocytosis/exocytosis (Table S5). Importantly, 92 SNPs were located on genes involved in Auxin, ABA and ethylene-activated and Jasmonic acid, gibberellic acid and sugar mediated signaling pathways; G-protein, apoptotic, cell surface receptor/Wnt signaling, signal recognition and transduction (Table S5). A further 63 SNPs were associated with genes from 35 different classes of TFs (Table S6). In total, 166 SNPs were located on genes with enzymatic activity (ligases, transferases, hydrolases), particularly kinases, which carried 77 SNPs. Some of these SNPs were closely related to post-translational modifications, such as phosphorylation and glycosylation, and to protein localization (Table S6). Genes involved in core cellular machineries, such as transcription, translation and replication, carried 198 SNPs (Table S7). Fifty-one SNPs were found within genes with Zn, Ca, Mg, Fe and metal ion binding activities, and 111 SNPs were associated with structural molecules, and thus, were part of different cellular organelles (Table S8). The highest number of SNPs (275) were confined to genes of various metabolic processes including photosynthesis, carbohydrate metabolism, oxidation-reduction, the Krebs cycle/respiration, protein catabolism, antioxidant activity, sugar, protein and lipid biosynthesis (Table S9).

**Table 5:** List of 44 ion transporters/channels annotated to polymorphic SNPs in a segregating wheat population developed for salt tolerance.

SNP ID	IWGSC Sequence Hit	Blast Top Hit Spp.	Annotated Channel/Transporter	Transports
AX-94778362	lcl|Traes_4BL_5A58CACB2.1	*T*. *durum*	HKT transporter	K
AX-95224228	lcl|Traes_6BS_E420DDD6D.1	*H*. *vulgare*	K+-H+ exchange	K
AX-94995317	lcl|Traes_1DS_07F02E427.1	*A*. *tauschii*	K transporter 12	K
AX-94388980	lcl|Traes_5DS_49CF8A4C4.1	*H*. *vulgare*	K (+) efflux antiporter	K
AX-95215612	lcl|Traes_XX_D8915FD17.1	*A*. *tauschii*	Voltage-gated K channel	K
AX-95654644	lcl|Traes_1BL_611DF0433.1	*H*. *vulgare*	Jacalin-related lectin 3	K
AX-94699167	lcl|Traes_5AL_51E31BF07.1	*T*. *urartu*	Out-rectifying K channel	K
AX-94484138	lcl|Traes_3B_81DB429AC.1	*T*. *urartu*	Chloride channel CLC-e	Cl
AX-94546397	lcl|Traes_2DL_A591AC867.1	*A*. *tauschii*	Chloride channel CLC-g	Cl
AX-95126745	lcl|Traes_4BL_E0ABA8471.1	*A*. *tauschii*	Cation Ca exchanger 4	Ca
AX-95069958	lcl|Traes_1AS_429D67C42.1	*A*. *tauschii*	Ca stress-gated channel 1	Ca
AX-94849975	lcl|Traes_7BL_13D715DB0.1	*T*. *urartu*	Ca homeostasis ER	Ca
AX-94662401	lcl|Traes_4BS_06DC8C269.1	*A*. *tauschii*	Ca-transporting ATPase	Ca
AX-94635693	lcl|Traes_XX_0593C741B.1	*H*. *vulgare*	Zn transporter 6	Zn
AX-94414919	lcl|Traes_1DS_C2EFEFBB9.1	*T*. *aestivum*	Zn transporter 7	Zn
AX-95172326	lcl|Traes_1DS_D28FA6FF2.1	*A*. *tauschii*	Zn transporter At3g08650	Zn
AX-94495517	lcl|Traes_XX_79F99051D.1	*H*. *vulgare*	Metal tolerance C2	Zn
AX-95634832	lcl|Traes_1AS_0B179C27B.1	*T*. *urartu*	IQM1	Zn
AX-94755145	lcl|Traes_2DL_5C445EE47.1	*H*. *vulgare*	Mg transporter NIPA3	Mg
AX-95159756	lcl|Traes_2AL_065DBAB56.1	*H*. *vulgare*	Mg transporter NIPA4	Mg
AX-94692118	lcl|Traes_2BL_D5156A4A5.1	*H*. *vulgare*	Mg transporter NIPA4	Mg
AX-94624155	lcl|Traes_4AL_7541D0C33.1	*T*. *urartu*	ER membrane body 2-X2	Mg, Fe
AX-95142803	lcl|Traes_2DL_CCAE7B431.1	*A*. *tauschii*	Sulfate transporter	Sulfate
AX-94518655	lcl|Traes_3AL_224FB10D3.1	*T*. *aestivum*	Sulfate transporter	Sulfate
AX-94852973	lcl|Traes_XX_7D456E213.1	*T*. *urartu*	Nitrate transporter	Nitrate
AX-94991110	lcl|Traes_2BL_0E87D8729.1	*A*. *tauschii*	NRT1 PTR FAMILY	Nitrate
AX-95216700	lcl|Traes_2BS_88803DFE6.1	*A*. *tauschii*	NRT1 PTR FAMILY	Nitrate
AX-94863332	lcl|Traes_3B_91715BB56.1	*A*. *tauschii*	Anion transporter 7	Anions
AX-94775993	lcl|Traes_2BL_007AADDF1.1	*A*. *tauschii*	G-3-Phosphate transporter1	Anions
AX-94550729	lcl|Traes_4BL_32F50466D.1	*T*. *urartu*	Mo-anion transporter	Anions
AX-94583481	lcl|Traes_3DS_50B54D1FC.1	*A*. *tauschii*	WPP domain-associated	Cations
AX-94752371	lcl|Traes_2DL_51FF05F66.1	*A*. *tauschii*	Cu-transporting HMA5	Copper
AX-94936984	lcl|Traes_7DS_D439AB891.1	*H*. *vulgare*	Pyrophosphate H+ pump	Proton Pump
AX-94384299	lcl|Traes_XX_796D903AA.1	*H*. *vulgare*	ATP synthase Mitochon.	Proton
AX-95118708	lcl|Traes_7DL_41A6D7A34.1	*A*. *tauschii*	Cytochrome-c oxidase	Proton
AX-94486290	lcl|Traes_3AL_06CDB999D.1	*G*. *hirsutum*	ATP synthase Mitochon.	Proton
AX-94982994	lcl|Traes_2DL_B4C9A5695.1	*B*. *distachyon*	H+-exporting ATPase	Proton
AX-94713620	lcl|Traes_XX_DFFB37624.1	*B*. *distachyon*	Anthranilate BTase 1	Non-selective
AX-94909932	lcl|Traes_5AS_D7A8B1D1B.1	*H*. *vulgare*	Mechanosensitive channel	Ions
AX-95257567	lcl|Traes_2DL_C065A5C4A.1	*A*. *tauschii*	Solute carrier 22–15	Metal ions
AX-94985111	lcl|Traes_7AS_705BE4B61.1	*T*. *urartu*	Solute carrier fam 35-F1	Metal ions
AX-94757270	lcl|Traes_1AL_CEA78C84D.1	*T*. *urartu*	S deficiency-induced 1	S
AX-94560970	lcl|Traes_1BL_6741F0C8B.1	*T*. *urartu*	S deficiency-induced 1	S
AX-94869513	lcl|Traes_1DL_36CEA53FD.1	*A*. *tauschii*	S deficiency-induced 1	S

Furthermore, 92 SNPs were associated with growth-related processes, including cell division/growth, meristem growth, autophagy, apoptosis, cell wall biogenesis/organization, lateral root development, leaf and shoot morphogenesis, flowering time; and flower, pollen, ovule and embryo sac development, while 48 more were located on genes related to stress responses, including disease resistance, drought, wounding, toxicity and freezing tolerance (Table S10). Finally, 109 SNPs were linked to hypothetical or uncharacterized proteins (Table S10).

### *In-silico* expression analysis of candidate genes

Gene expression was categorised into six groups: up-regulated, expression not conclusive, down-regulated, expressed under salinity only, data not conclusive and no expression with 122, 425, 136, 156, 241 and 209 genes falling into these groups respectively (Table S11).

## Discussion

### The Wheat 35 K array is suitable for construction of genetic linkage map

We have constructed a high-density SNP linkage map of bread wheat using the wheat 35 K array, which consisted of probes for 35,143 exome-captured SNPs. Of these SNPs, 46.1% or 16,210 were monomorphic in our population and only ‘PHR’ SNPs (3,381 or 9.6%) were used for linkage map construction contrary to the recent paper^19^ which used ‘NMH’ and ‘OTV’ SNPs as well. This was because we genotyped an F_2_ population, so only PHR SNPs could be tested for typical F_2_ segregation. Removal of markers with segregation distortion/bias is essential for getting a good quality linkage map, which was performed by Chi-square test with sequential Bonferroni correction^30^. From the 1072 SNPs passing the test, 988 were assigned to 21 chromosomes. Only 84 polymorphic markers were assigned to the D genome as compared to 342 and 562 markers assigned to A and B genome. This has been linked to lower nucleotide diversity of the D genome due to its relatively recent evolutionary origin^37,38^. Our genetic map has a total length of 2317.88 cM in comparison to 3739.23 cM for the published wheat consensus map^19^, largely due to the lack of diversity/segregation in the D genome under salinity. Of 988 SNPs, 398 SNPs were assigned to chromosomes for the first time, and 79 markers were assigned to different chromosomes from the consensus map. These novel and conflicting markers show genetic diversity originating from genetic differences between European and Pakistani wheat lines. Secondly, we used an F_2_ population instead of inbred lines, which may have contributed in different segregation patterns. Even so, the large majority (511 of 590) of markers common to both maps were assigned to the same chromosomes^19^.

### Several novel and major QTLs were mapped for minerals and salt tolerance

We mapped 49 QTLs for 22 traits under salt stress on 12 chromosomes, including four QTLs on two chromosomes of the D genome. Salt tolerance in wheat is mainly conferred by NAX or reduced Na^+^ uptake, as Na^+^ influx leads to reduce photosynthesis, growth, development and yield^5,7,9^. The genetic basis of NAX was unknown until the identification of a major NAX QTL on chromosome 2A^39^; and QTL mapping for salt tolerance has focused on NAX in recent years^23,25,26^. Among the six mapped RNAX and SNAX QTLs, two closely located QTLs (qSNAX.2A.1 and qSNAX.2A.2) and qRNAX.2A.1 coincided with the major NAX locus *Nax1* or *HKT1;4* in durum wheat^39^ and three NAX QTLs found on 2A in bread wheat^25^. Similarly, the qRNAX.6A.2 QTL has also been reported previously^25,26^. We mapped two novel and major QTLs (qSNAX.7A.3 and qRNAX.7A.3) on chromosome 7A, which contributed 13.69 and 15.35% of the phenotypic variation of RNAX and SNAX and accounted for 11.23 and 19.79% of the observed salt tolerance (DRW and DSW respectively). The HKT genes are well characterized regulators of K^+^ and/or Na^+^ transport in plants and encode proteins that reduce Na^+^ transport to the shoot, thus conferring salt tolerance^5^. Accordingly, QTL mapping for K^+^ conc. under salinity has also been performed by some groups^23,25–27^. We identified a major SKC QTL on 6A contributing 7.46 and 9.76% of the phenotypic variation in K^+^ conc. and salt tolerance. Another novel and major QTL (qRKC.4B.2) presented 11.31% of phenotypic variation of RKC. Another RKC QTL mapped on 3D was consistent with a reported QTL^25^ and the remaining SKC and RKC QTLs were co-located with the SNAX and RNAX QTLs. This co-localization of QTLs is explained by the functional correlation between these traits.

Due to the focus on NAX and K^+^ QTLs, to our knowledge the genetics of Ca^2+^ and Mg^2+^ accumulation under salinity has only been investigatedonce^27^. We mapped two major QTLs for RCalC (qRCalC.6B.3 and qSCalC.6B.2), which contributed 5.92 and 11.87% of salt tolerance and 10.91 and 6.52% of Ca phenotypic variation. The above mentioned and two RCalC QTLs on 3B coincided with previously reported QTLs^27^, but a novel QTL on 6A also presented 8.98% of the observed SCalC phenotypic variance. Among Mg^2+^ (RMgC and SMgC) QTLs, a 2A QTL presented 6.37% of SMgC phenotypic variation and qSMgC.6B.2 contributed 8.36% to salt tolerance. Another novel QTL on 5A accounted for 5.58% of salt tolerance. We also mapped 27 novel QTLs for P, Zn, Fe, Mn, Cu, S and Boron concentrations in wheat root and shoot under salinity for the first time as QTLs for these mineral were previously reported under different water regimes only^24^. Among them, the most important major QTL for RZnC on 7A contributed 11.23 and 12.08% to salt tolerance and RZnC phenotypic variation. One each of the FeC and MnC QTLs, found on 6B, presented 5.92% of salt tolerance and the Mn QTL contributed 14.16% to Mn phenotypic variation. Similarly, a major qRFeC.6A.2 contributed 12.96% of Fe phenotypic variation. Despite the low contribution of Boron, P and S QTLs to salt tolerance, their contribution to mineral phenotypic variation under salinity was high, which could be useful information for future wheat breeding.

We found five QTL clusters on the A and B genome containing QTLs for several minerals. Such clusters are expected as changes in cellular Na^+^ could affect concentrations of many other ions; indeed, previous studies have shown clustering of QTLs for closely correlated salt traits^23,24,26^. Each cluster may represent either a single gene or several closely linked genes; e.g. the occurrence of two closely linked NAX QTLs on 2A and the co-localization of Zn and NAX QTLs. This co-localization or QTL clustering is due to high the correlation coefficient among the traits of the respective QTLs as highlighted (Table S4).

### Functional annotation of segregating SNPs highlighted complexity of salt tolerance

Segregating F2 populations are valuable material for the dissection of the genetic architecture of complex traits, as they depict maximum segregation in phenotype and polymorphism at molecular level^14,39^. The Wheat 35 K Array contains exome-captured SNPs; thus, flanking sequences of segregating SNPs could be used for functional annotation, which revealed that genes of various biological processes and molecular functions are likely to be involved in salt tolerance mechanisms. SNP-carrying sequences were highly similar to those from *A*. *tauschii* and *T*. *urartu*, reflecting their close ancestral relationships with bread wheat^37^. Another top species from BLAST comparisons was *H*. *vulgare*, the most salt tolerant cereal^15^; indicting shared tolerance genes between wheat and barley. We annotated 44 ion transporters responsible for ion homeostasis. Among the seven annotated K transporters/channels, the role of HKTs i.e. TmHKT1;5-A *Nax2*, HKT1;4 *Nax1* as sodium excluders^5,25^, K^+^/H^+^ exchanger, TaNHX2, as K^+^/H^+^ antiporter^40^ and Jacalin-related lectin 3 (TaJRL3) in salt tolerance response^41^ was reported previously. The K^+^ outward-rectifying channel (KORC) negatively regulates salt tolerance by K^+^ efflux from plant roots under salinity^42^, but the functions of the remaining K^+^ channels are not known. The annotated chloride channels (CLC-g and CLC-e) could be linked to previously mapped Cl^−^ QTLs including a major 5A QTL^27^. The annotated Pyrophosphate-energized proton pump (H^+^-PPase or TVP1) and four proton transporters were previously found to induce the sequestration of Na^+^ ions into the vacuole and act as Na+/H+ antiporters to confer salt tolerance in wheat^43^. However, none of the four Ca, five Zn transporters, three nitrate transporters and two each of Mg, Sulfate and Cu-transporters have been characterized for their salt tolerance response in wheat. Another 50 SNPs annotated for transport of proteins, mRNA, glucose, malate, fatty acids and proline need further investigation under salinity.

Understanding of complex abiotic stress signalling pathways is essential for a successful breeding program^34,44^. In total 92 SNPs were located within genes involved in several signalling pathways, e.g. signal recognition, transduction, cell surface receptor and Wnt signalling to identify stress stimuli. Annotation of apoptotic signalling genes could be linked to the hypersensitive response to avoid stress injury^45^. Similarly, a total of 14 genes were identified for ABA and ethylene activated signalling and for JA and SA-mediated signalling pathways, which are thought to be involved in conferring salt tolerance^3^. Moreover, some genes for ABA, ethylene and JA signalling also confer salt tolerance in wheat^46,47^. We identified 22 genes for auxin biosynthesis, transport and auxin-activated signalling pathways, but the role of auxin signalling under salinity is not known. TFs are proteins that regulate the expression of several stress-related genes^34^ and 63 annotated SNPs were linked to 35 classes of 52 TFs e.g. MYB44, WRKY16, WRKY70, bZIP17, NAC17, NAC78 etc. However, bHLH140, GATA26, ZNFX1-NFXL1, EIN3, ABI3, ARF3, ARF5 and ARF21 appear to be major salinity responsive TFs based on their GO annotations. Only the TFs ABI3 and ARF3 have previously been reported to be involved in salt tolerance mechanisms and signalling^46,48^ in wheat.

Interestingly, 198 SNPs were located on genes involved in various nucleic acid processes including chromatin modifications, helicases, DNA repair mechanisms, DNA replication, transcription and translation regulation. Some of these genes were over-expressed under salinity^3^. Other such gene groups coded for epigenetic (DNA, tRNA, rRNA and histone-lysine methylation) or epi-transcriptomic (splicosomal complexes, splicing site recognition, mRNA splicing, mRNA based gene silencing) processes. As these reactions are core cellular functions they may regulate the expression of salinity related genes^49^. Additionally, 166 SNPs were located on genes with enzymatic (ligases, transferases, hydrolases, isomerase and kinase) or protein modification activity. These included 77 kinases, protein kinases and protein serine/threonine kinases coding for protein phosphorylation or post-translational protein modification. Protein serine/threonine kinases and protein kinases play a role in wheat salt tolerance and ABA signaling respectively^48,50^. Genes for other post-translational processes (protein de-phosphorylation, dimerization and glycosylation) were also identified. Another 51 SNPs were linked to genes involved in Zn, Ca, Mg, Fe and metal ion binding, as metal ions are part of several enzymes that may have direct roles in conferring salt tolerance.

The largest number of annotated SNPs, 275, were linked to genes of metabolic processes. The biggest subgroup consisted of 65 SNPs associated with photosynthesis regulation, chloroplast fission/organization, chlorophyll biosynthesis/catabolism, Photosystem (PS) I and II complex, PS II assembly, light reaction, Carbon/energy pathway, chloroplast DNA synthesis/translation; and synthesis of photosynthetic sugars (glucose, galactose, fructose, mannose). The role of these genes in photosynthesis under salinity in wheat has not been thoroughly investigated, but 22 photosynthetic proteins showed differential expression under salinity in wheat^51^. Additionally, 45 genes were identified for oxidation-reduction (Redox) processes, which are the backbone of cell mechanisms. A redox gene, 12-oxophytodienoate reductase 1 (OPR1), confers salt tolerance to wheat by enhanced ABA signalling and reactive oxygen species (ROS) scavenging^52^. The redox genes coding for peroxidase 1, 2 and 12, and 19 genes for synthesis of antioxidants (glutathione, flavonoid, carboxylic acid, lactate, cytokinin, vitamin B and E) could be involved in ROS scavenging under salinity induced osmotic stress. Genes for ubiquitin, proteasome and proteolysis dependent protein catabolism could destroy the unwanted proteins under salinity. However, roles of 21 respiratory genes (photorespiration, respiratory chain complex I and II, glycolysis, and Kreb cycle) and 64 genes for biosynthesis of carbohydrates, lipids and proteins needs to be investigated. Among the 92 SNPs associated with genes of several growth stages such as root/shoot development to reproductive growth (flowering time, pollen germination, ovule development etc.), inheritance of flowering time under salinity has been reported^15^.

### Several annotated genes were transcriptionally expressed

In order to validate the role of annotated SNPS/genes in conferring salt tolerance, they were aligned with published transcriptome data describing genes that are differentially expressed under salinity^3^. A total of 122 of the annotated genes were up-regulated *in silico*, which included protein, mRNA, nitrate and cations transporters; several Wnt/ABA/auxin activated signaling molecules; signal transduction, kinases, proteolysis, REDOX process, flavonoid metabolism, defense response, cell wall/xylum development genes etc. On the other hand, 136 genes were down-regulated under salinity. The only available salt-expressed transcriptome in wheat root is based on a single genotype; therefore, several of the annotated genes, such as photosynthetic genes, were not expected to show differential expression.

### Conclusions and prospects

We have identified two novel major QTLs on wheat chromosome 7A which contributed 11.23 and 15.79% to salt tolerance, and 13.69–15.35% to NAX in our population. Another major Zn QTL contributed 12.08 and 11.23% to Zn phenotypic variation and salt tolerance respectively. A major SCaIC QTL also accounted for 11.87% of the observed salt tolerance trait. We have also identified other novel QTLs which contributed 10.91, 12.96,11.31 and 14.16% of Ca, Fe, K and Mn phenotypic variation. Other mapped QTLs represented 2.1–8.98% of phenotypic variation of different minerals. These novel QTLs could be used for could be used for MAS breeding for salt tolerance and breeding for biofortification of wheat for Ca, Zn, Mg, Fe and Mn. We have also annotated 1293 segregating SNPs, which were located within genes for various ion channels, signalling pathways, TFs, metabolic pathways etc. and 258 of them were differentially expressed under salinity, indicating that they may have a role in salt tolerance. As the published transcriptome used for this analysis is based on Roche 454-GS FLX sequencing, future transcriptome data fromsalt stressed wheat using Illumina technology could help to further understand gene expression under salinity. The characterization of the annotated genes described here will help to dissect salt tolerance mechanisms, guiding future breeding for this important trait.

## Electronic supplementary material

Supplementary File
