# Author Correction: Mapping QTLs conferring salt tolerance and micronutrient concentrations at seedling stage in wheat

**DOI:** 10.1038/s41598-020-75482-y

**Published:** 2020-10-22

**Authors:** Babar Hussain, Stuart James Lucas, Levent Ozturk, Hikmet Budak

**Affiliations:** 1 Faculty of Engineering and Natural Sciences, Sabanci University, Istanbul, Turkey; 2 SU Nanotechnology Research and Application Centre, Sabanci University, Istanbul, Turkey; 3 Cereal genomics Lab, Department of Plant Sciences and Plant Pathology, Montana State University, Bozeman, MT USA

Correction to: *Scientific Reports* 10.1038/s41598-017-15726-6, published online 15 November 2017

This Article contains an error in the order of the Figures. Figures 3 and 4 were published as Figures 4 and 3, respectively. The correct Figures 3 and 4 appear here as Figures 1 and 2, respectively. The Figure legends are correct.

**Figure 1:**
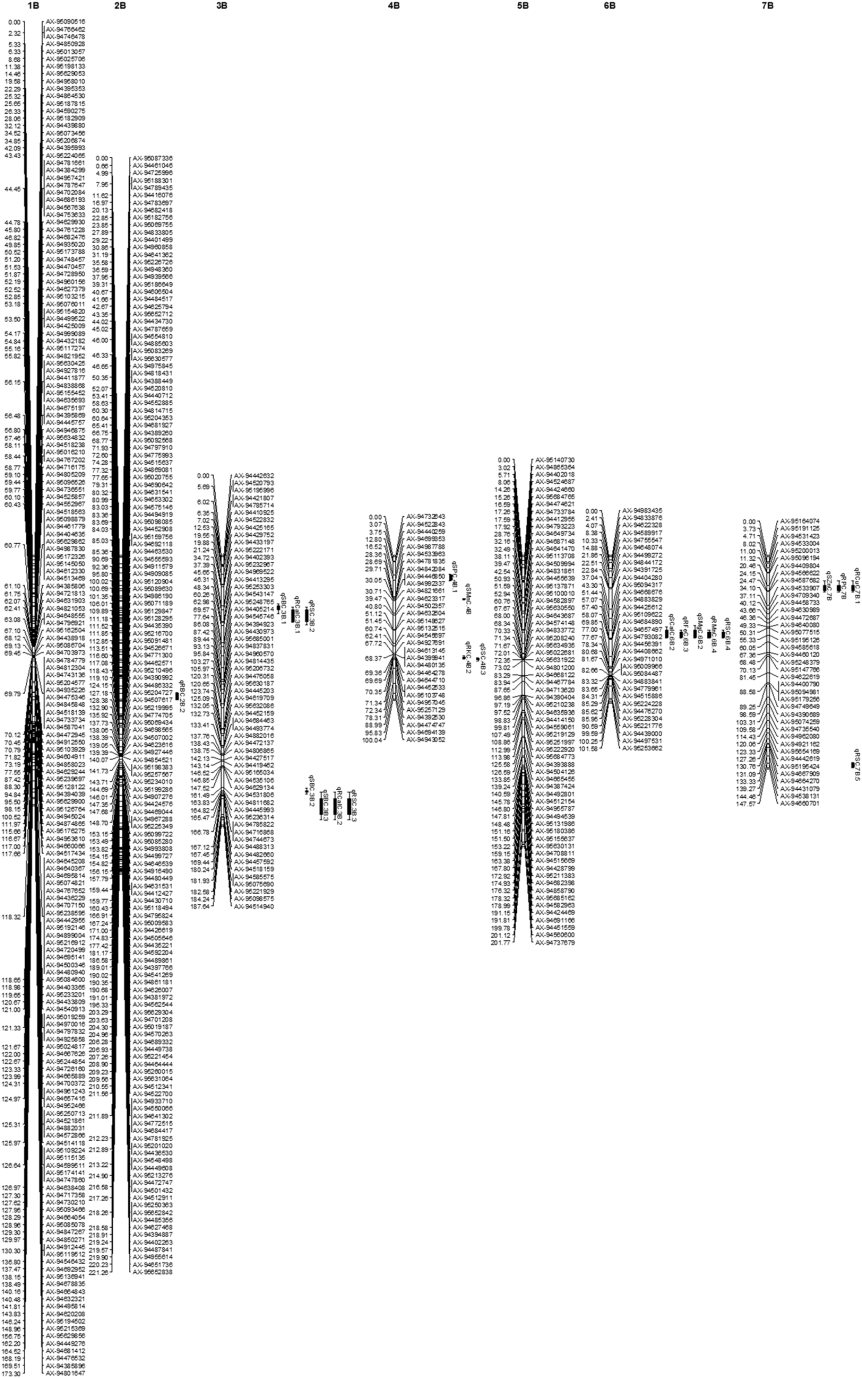
Additive QTLs mapped on B genome for salt tolerance traits and mineral concentrations under salt stress in wheat F_2_ population.

**Figure 2:**
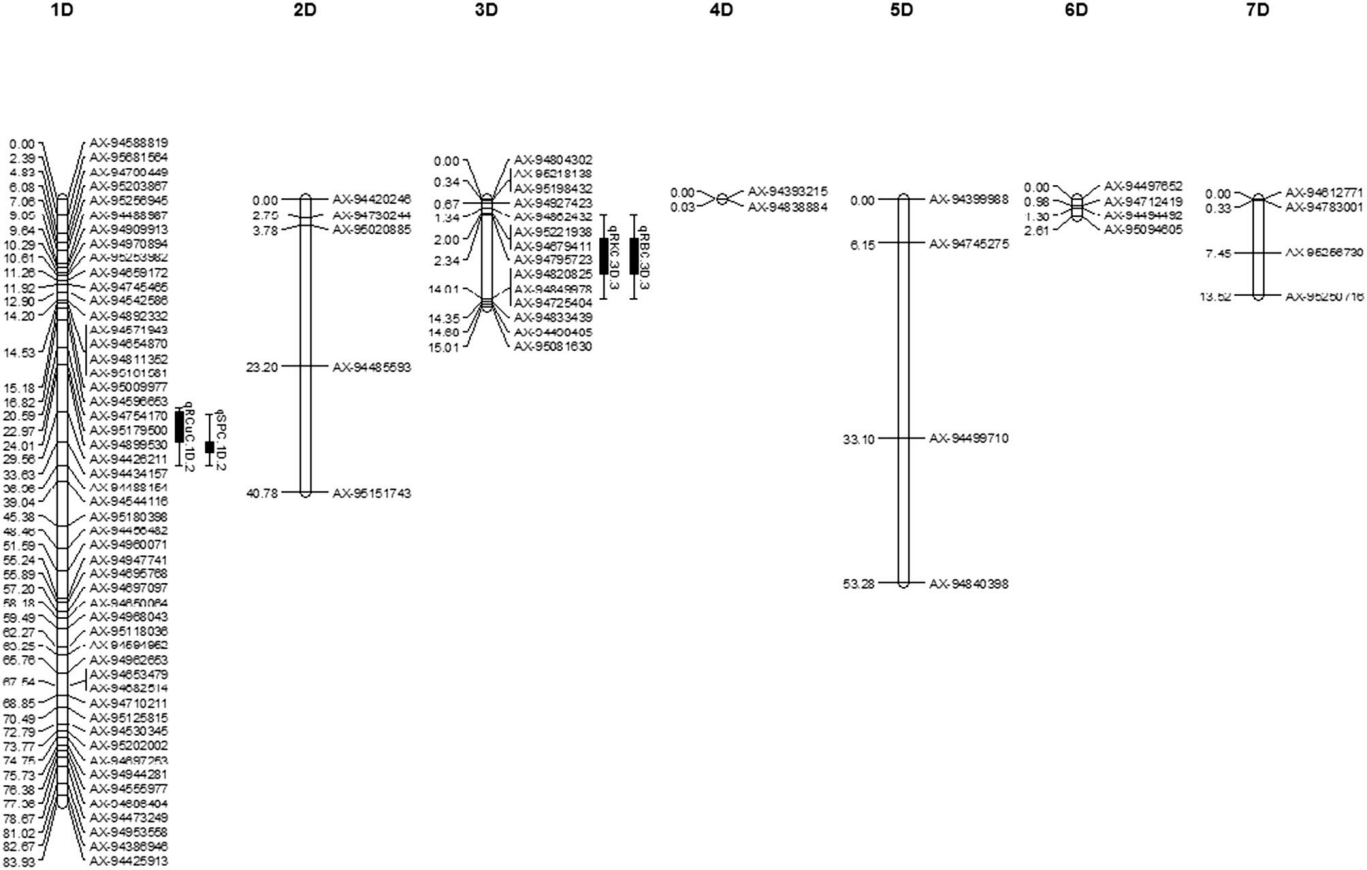
Additive QTLs mapped on D genome for salt tolerance traits and mineral concentrations under salt stress in wheat F_2_ population.

